# Mapping the NASH-CHECK to the EQ-5D-5L in a MASLD population in seven European countries

**DOI:** 10.1186/s12955-026-02554-4

**Published:** 2026-08-12

**Authors:** Tomos Robinson, Hangjian Wu, Elisabetta Bugianesi, Helena Cortez-Pinto, Lynda Doward, Mathias Ekstedt, Adriaan G. Holleboom, Yemi Oluboyede, Vlad Ratziu, Manuel Romero-Gomez, Clifford Brass, Kristy Wonders, Luke Vale, Quentin M. Anstee

**Affiliations:** 1 Population Health Sciences Institute, Faculty of Medical Sciences, Newcastle University, Newcastle Upon Tyne, UK; 2 NIHR Applied Research Collaboration North East and North Cumbria, Newcastle-Upon-Tyne, UK; 3 Division of Gastroenterology, AOU Città della Salute e della Scienza University of Turin, Turin, Italy; 4 Clínica Universitária de Gastrenterologia, Faculdade de Medicina, Universidade de Lisboa, Lisbon, Portugal; 5 RTI Health Solutions, Manchester, UK; 6 Department of Gastroenterology and Hepatology, Linkoping University, Linkoping, Sweden; 7 Department of Health, Medicine and Caring Sciences, Linkoping University, Linkoping, Sweden; 8 Amsterdam University Medical Center, Amsterdam, Netherlands; 9 University of Amsterdam, Amsterdam, Netherlands; 10 Putnam PHMR, London, UK; 11 Sorbonne University, Paris, France; 12 Catedrático de Medicina, University of Seville, Seville, Spain; 13 Resolution Therapeutics, London, UK; 14 Translational & Clinical Research Institute, Faculty of Medical Sciences, Newcastle University, Newcastle Upon Tyne, UK; 15 London School of Hygiene and Tropical Medicine, London, UK; 16 Newcastle NIHR Biomedical Research Centre, Newcastle Upon Tyne Hospitals NHS Trust, Newcastle Upon Tyne, UK

## Abstract

**Background:**

Metabolic-dysfunction associated steatotic liver disease (MASLD) is a progressive chronic liver condition characterised by substantial inter-patient variation in disease severity, with the probability of progressing to hepatic fibrosis, cirrhosis or hepatocellular carcinoma. The prevalence of MASLD is increasing rapidly and may have a significant impact on health-related quality of life (HRQoL). A novel non-preference-based Patient-Reported Outcome Measure (PROM) called NASH-CHECK has been developed to assess outcomes in metabolic dysfunction-associated steatohepatitis (MASH), previously called non-alcoholic steatohepatitis (NASH). Preference-based outcome measures are needed to generate quality adjusted life years (QALYs) for economic evaluation. The aim of this study was to generate mapping algorithms to predict EQ-5D-5L utility scores from the NASH-CHECK in a MASLD population.

**Method:**

A large dataset comprising 1,971 MASLD cases from seven European countries (France, Italy, Portugal, Netherlands, Spain, Sweden and UK) was used to generate the mapping algorithms. Regression results from a number of popular candidate models were compared against each other in terms of statistical and visual fit, including more conventional models (OLS, Tobit, Two-Part) and the more recently developed Adjusted Limited Dependent Variable Mixture Model (ALDVMM). Mapping was conducted separately for each of the seven countries.

**Results:**

The ALDVMM with higher numbers of components provided the best visual fit across all samples, especially at the low end (representing very poor health states and worse-than-death health states) and high end (representing fairly good health states and full health) of the EQ-5D-5L distributions. The ALDVMM also produced predicted estimates which had the best statistical fit (in terms of mean absolute error, root mean square error and information criteria where applicable) for samples with relatively large number of observations.

**Conclusions:**

Using an ALDVMM with higher numbers of components provides estimates across the whole distribution for the prediction of EQ-5D-5L as a function of NASH-CHECK scores for people with MASLD in several European countries. This enables QALYs to be indirectly calculated in this patient population when a preference-based measure such as the EQ-5D-5L has not been collected.

**Supplementary Information:**

The online version contains supplementary material available at https://doi.org/10.1186/s12955-026-02554-4.

## Background

Metabolic-dysfunction associated steatotic liver disease (MASLD, formerly called non-alcoholic fatty liver disease [NAFLD]) is a progressive chronic liver condition characterised by increased fat accumulation (metabolic-dysfunction associated steatotic liver, MASL) and hepatic inflammation (metabolic-dysfunction associated steatohepatitis, MASH) [1]. Disease natural history is highly dynamic with substantial inter-patient variation in disease severity and outcomes but many patients exhibit progressive hepatic fibrosis and may progress to cirrhosis and/or hepatocellular carcinoma [2, 3]. The prevalence of MASLD is increasing rapidly and is challenging healthcare systems worldwide as it consumes increasing resources [4].

The NASH-CHECK is a novel Patient-Reported Outcome Measures (PROM) developed to assess symptoms and health related quality of life (HRQoL) related impacts of MASH. The measure facilitates understanding of the symptoms and wider impact of MASH from the patient perspective and would complement data on key clinical endpoints in MASH. Details regarding the development and validation of the NASH-CHECK are reported in Doward *et al.,* (2020) [5] and Twiss et al. (2023) [6]. Recent work has evaluated the content validity of the NASH-CHECK for patients with compensated cirrhotic, demonstrating that the NASH-CHECK is a suitable PROM in this patient population [7].

Evaluation of medicines and health care decision making requires an understanding of the clinical and patient-perceived value of treatment, and the economic value of treatments. Disease-specific PROMs capture detailed, condition-relevant outcomes but do not directly provide utility values required for economic evaluations like cost-utility analysis. While generic, preference-based measures such as the EQ-5D-5L can be used to derive health-state utility data, they lack the precision and relevance to evaluate outcomes in a given health condition. For example, concepts important to patients with MASH such as fatigue may be poorly captured by generic measures. Preference-based measures also differ from non–preference-based measures in the way the scoring algorithms are derived. They are estimated from the values that people place on different aspects of health rather than a simple summative scoring procedure [8]. In line with recommendations from a number of health technology assessment bodes (such as NICE in England and Wales), a preference-based outcome measure is needed to generate quality adjusted life years (QALYs), a generic measure of disease burden commonly used in economic evaluation such as cost-utility analysis (CUA). Mapping utility values from disease-specific PROMs combines the depth of patient-relevant, condition-specific outcomes with the economic standardisation needed for the derivation of QALYs, offering a more complete and meaningful assessment of treatment value, with the efficiency of administering a single PROM to the patient.

The aim of this study was to develop an algorithm that can predict EQ-5D-5L utility scores from the NASH-CHECK in a MASLD population. The EQ-5D-5L is a generic, preference-based PROM widely used to measure health status as part of CUAs. A mapping algorithm reflects the statistical relationship between the preference and non–preference-based instruments and so requires access to data where both instruments have been collected simultaneously [9]. Mapping studies have been successfully adopted and implemented in many other clinical areas, including cancer, cardiovascular disease and musculoskeletal disorders [10]. However, to the author’s knowledge no mapping studies have previously been conducted in hepatology research. Such an approach enables economic evaluations seeking to support payers and other decision-makers evaluating interventions for MASLD, where preference-based measures such as the EQ-5D-5L have not been directly collected. In addition, the large-scale data collection covers seven European countries (France, Italy, Netherlands, Portugal, Spain, Sweden, UK), allowing the prediction algorithms to be used in a wide range of geographical areas where an economic evaluation for MASLD related disease is to be conducted.

## Methods

### Data

This study was conducted in accordance with the MAPS (MApping onto Preference-based measures reports Standards) checklist [11] and the ISPOR guidance for outcome research task report [12]. To develop a mapping algorithm from the NASH-CHECK to the EQ-5D-5L in a MASLD population, we used data from consenting participants from the European Steatotic Liver Disease (SLD) Registry [13]. The European SLD Registry is the largest international cohort of patients with histologically characterised MASLD and contains data from patients recruited across European countries and the USA. As well as the NASH-CHECK and EQ-5D-5L, the registry also contains demographic information (such as age and gender) that could be used to inform the mapping algorithm. It also included a range of clinical data, including anthropomorphic measures, comorbidities and the results of routine clinical investigations (haematology, clinical biochemistry), as well as a rich biomarker dataset [14]. These clinical measures were not considered for use in the mapping algorithms. However, some comorbidities (for example the presence of type 2 diabetes or dyslipidaemia) are included in the descriptive analysis of the samples, along with a measure of liver fibrosis.

The date of the data download from the European NAFLD Registry was 2^nd^ October 2023, with data collected between January 2017 and September 2023. The original intention was to generate separate mapping algorithms for each country for which data was available. However, mapping is data intensive, with larger sample sizes required to reduce the prediction error in the model. Full data on both the NASH-CHECK and the EQ-5D-5 L were available for at least 100 patient observations in seven countries (France, Italy, Netherlands, Portugal, Spain, Sweden, the UK). Therefore, mapping algorithms were calculated for these seven countries only, with countries such as Germany, Finland and the USA excluded for having too few observations. It is worth emphasising that this refers to the number of *observations* in each country sample rather than the number of individual participants. In all countries there were some respondents with multiple observations, further details are provided in Sect. “Descriptive analysis”.

### NASH-CHECK & EQ-5D-5L

The NASH-CHECK was scored in accordance with the published scoring guidelines [15]. The NASH-CHECK comprises 28 items. Five of the items (Abdominal Pain, Abdominal Bloating, Fatigue, Sleep and Itchy Skin) are converted into ‘Single Symptom Item Scales’ and the four items assessing cognitive symptoms are used to calculate the ‘Cognitive Symptom Scale’. The remaining items are used to calculate the three HRQoL scale scores: the ‘Activity Limitations Scale’ (8 items), ‘Emotional Impact Scale’ (4 items) and ‘Social Impact Scale’ (7 items). All single item and scale scores range from 0 to 10, with higher scores indicating greater impairment on the underlying construct.

The EQ-5D-5L covers five dimensions (Mobility, Self-Care, Usual Activities, Pain/Discomfort and Anxiety and Depression), with each dimension having five response levels (No Problems, Slight Problems, Moderate Problems, Severe Problems and Extreme Problems) [16]. The responses to the EQ-5D-L were converted into utility scores using previously developed country-specific algorithms as described below.

### EQ-5D-5L value sets

For the French, Italian, Dutch, Portuguese, Spanish, Swedish and UK samples, the responses to EQ-5D-5L were converted into utility scores using the country-specific value sets developed by Andrade *et al.,* (2020) [17], Finch *et al.,* (2022) [18], Versteegh *et al.,* (2016) [19], Ferreira *et al.,* (2019) [20], Ramos-Goni *et al.,* (2018) [21] Sun *et al.,* (2022) [22] and Rowen et al. (2026) [23] respectively.

### Regression models

The original intention was to use indirect (or ‘response’) mapping methods [24]. This indirect approach involves estimating the response levels to each of the individual dimensions of the target instrument (in this case the EQ-5D-5L) using ordered regression methods and then summing together these estimated responses. However, an inspection of the EQ-5D-5L data in the European SLD Registry found very small number of observations in the worst levels of the Mobility, Self-Care and Usual Activities dimensions. Indirect methods require a large sample size and enough responses at all levels for each dimension in order for the mapping model to predict all of the health states that can be generated. Consequently, indirect methods were not used in this study, and instead direct mapping methods (which map directly to a value set) were used.

Four regression models were used in the analysis: Ordinary Least Squares (OLS), Tobit, Logit-OLS ‘Two-Part’ Model and the Adjusted Limited Dependent Variable Mixture Model (ALDVMM). The OLS, Tobit and Two-Part models are commonly used methods that have been used in a wide variety of previous mapping studies [10]. The OLS model is commonly used as a starting method in mapping studies given its simplicity in the assumption of the dependent variable in regression analysis [25]. However, the distribution of the EQ-5D utility values are usually non-normal and censored at 1, and therefore more advanced regression models such as the Tobit and the Two-Part models are likely to be more appropriate given that these models consider the fact that there may be a mass of observations at the upper bound of the distribution [26, 27].

It has been argued however that despite taking into account the potential mass of observations at the extreme of the distributions, the Tobit and Two-Part models are still not adequately equipped to model the unique distribution of EQ-5D value sets. As mentioned previously, the value set can include a mass of observations at 1, a large gap between this mass point and the next feasible EQ-5D state and a bimodal distribution for the remaining health states with a high degree of skewness. In response to this, the ALDVMM has been proposed as a flexible alternative to model EQ-5D data and has previously been shown to perform better than the models traditionally used in this area [28]. This model is semiparametric mixture model of adjusted ‘tobitlike’ distributions, which considers that the population is likely to be made up of several ‘latent classes’ of respondents. The number of latent classes (or ‘components’) can be varied in an attempt to find the optimal model specification. For a full description of the ALDVMM please see Hernandez-Alvara & Wailoo (2015) [29]. To account for the fact the multiple observations could be observed from the same individual over time, standard errors were clustered at the individual level in all regression models.

### Model comparison

The models used in the analysis were compared using widely used measures of overall model fit, including the Mean Average Error (MAE), Root Mean Squared Error (RMSE), the difference between the mean predicted value by the mapping algorithm and the mean observed value in the dataset. Additionally, the successfully converged ALDVMMs with different number of mixture components were compared in terms of their Akaike Information Criteria (AIC) and Bayesian Information Criteria (BIC).

Visual measures of fit were also applied to complement numeric fit measures [28, 30]. The reason for using these visual methods is that summary measures such as the MAE and RMSE only show how the models fit on average but give no indication on how the model may fit at different parts of the distribution. Flexible model structures (such as the ALDVMMs) may fit better over the full distribution; however, this may not be reflected in the summary measures of model fit. Therefore, we produced graphs of the cumulative distribution functions (CDFs) for each of the models, as these graphs show how closely the predicted data fits the actual data over the full distribution.

## Results

### Descriptive analysis

The data used in this study included 1,971 observations from 1,373 individuals. From these 1,971 observations, 173 were dropped due to missing values for either the EQ-5D-5L or NASH-CHECK (a requirement for mapping), leaving a final estimation sample of 1,789 from 1,269 individuals. Descriptive statistics for the included samples used in the study are shown in Table 1 and refer to the individual responses rather than the number of individuals. As shown, on average the samples from the UK, Portugal and Sweden were older than those from the Netherlands, Portugal, Spain and Sweden. All samples had higher proportions of males than females, especially in the samples from the UK and Netherlands. The Italian sample on average had a lower BMI than the rest of the samples. The Italian and Netherlands samples were less likely to have diabetes, and the Netherlands sample was less likely to have hypertension. The UK sample tended to have high dyslipidaemia, whilst this rate was relatively low for the Italian and Spanish samples. In terms of liver fibrosis, the Italian, Portuguese and UK samples were more likely to have a confirmed case of cirrhosis (between 18 and 27%) compared to the other countries, where cases ranged from 6 to 12%.

**Table 1:** Descriptive statistics of final estimation sample by country

	France (n = 100)	Italy (n =473)	Netherlands (n = 112)	Portugal (n = 122)	Spain (n = 206)	Sweden (n = 114)	UK (n = 671)
**Age**	58.45 (12.77)	54.02 (12.62)	51.28 (13.72)	59.48 (13.83)	55.79 (11.75)	60.18 (11.54)	59.12 (12.40)
BMI *	33.46 (6.42)	30.46 (4.94)	32.44 (5.28)	32.74 (7.08)	34.42 (7.23)	32.03 (5.64)	33.97 (5.83)
**Gender** Female (%) Male (%) Missing (%)	40.00 60.00 0.00	41.86 58.14 0.00	34.82 65.18 0.00	63.11 36.89 0.00	50.49 49.51 0.00	43.86 56.14 0.00	37.56 62.42 0.00
**Diabetes** Yes (%) No (%) Missing (%)	51.00 49.00 0.00	38.48 61.52 0.00	43.75 56.25 0.00	57.38 34.43 8.20	49.03 50.97 0.00	64.04 35.96 0.00	64.72 35.18 0.30
**Hypertension** Yes (%) No (%) Missing (%)	66.00 34.00 0.00	54.54 45.45 0.00	38.39 61.61 0.00	59.02 32.79 8.20	57.77 42.23 0.00	65.79 34.21 0.00	60.06 39.34 0.60
**High Triglycerides** Yes (%) No (%) Missing (%)	21.00 79.00 0.00	30.23 69.77 0.00	26.79 73.21 0.00	34.43 54.92 10.66	32.53 65.05 2.43	28.95 71.05 0.00	53.06 45.75 1.19
**High Cholesterol** Yes (%) No (%) Missing (%)	48.00 52.00 0.00	39.75 60.25 0.00	45.54 54.46 0.00	39.34 50.82 9.84	32.52 62.62 4.85	49.12 50.88 0.00	49.78 49.48 0.75
**Confirmed Cirrhosis** Yes (%) No (%) Missing (%)	6.00 94.00 0.00	18.81 81.19 0.00	6.25 93.75 0.00	25.41 74.49 0.00	12.62 87.38 0.00	5.26 94.74 0.00	27.42 72.58 0.00

**Notes:** Means and standard deviations presented for continuous variables. Percentages presented for categorical variables

*1 missing BMI value in French sample, 82 missing BMI values in the Italian sample, 34 missing BMI values in the Portuguese sample, 3 missing values in the Spanish sample, 18 missing BMI values in UK sample

The responses to the EQ-5D-5L for the seven samples are shown in Table 2. As shown, the UK sample were more likely to report more severe problems than the other countries across all dimensions. For example, they were less likely to report ‘No Problems’ in all five EQ-5D-5L dimensions and more likely to report ‘Extreme Problems’ in three of the five dimensions. The responses from the Netherlands, Italian, Spanish and Swedish samples were broadly similar for the responses to the ‘Mobility’, ‘Self-Care’, ‘Usual Activities’ dimensions. The Netherlands sample were more likely to report ‘Slight Pain’ and ‘Moderate Pain’, but less likely to report severe anxiety problems.

**Table 2:** EQ-5D-5L responses by item response level in final estimation sample by country

	France (n = 100)	Italy (n = 473)	Netherlands (n = 112)	Portugal (n = 122)	Spain (n = 206)	Sweden (n = 114)	UK (n = 671)
**Mobility**							
No Problems	83.00%	69.98%	73.21%	53.28%	67.96%	71.05%	49.78%
Slight Problems	5.00%	17.34%	11.61%	22.13%	17.96%	20.18%	18.78%
Moderate Problems	7.00%	10.15%	11.61%	17.21%	12.14%	7.02%	19.08%
Severe Problems	5.00%	2.33%	3.57%	7.38%	0.97%	0.88%	11.48%
Unable to Walk	0.00%	0.21%	0.00%	0.00%	0.97%	0.88%	0.89%
**Self-Care**							
No Problems	96.00%	90.70%	89.29%	72.95%	85.44%	86.84%	73.62%
Slight Problems	2.00%	6.34%	4.46%	15.57%	10.68%	8.77%	13.26%
Moderate Problems	2.00%	2.33%	6.25%	9.02%	3.40%	3.51%	8.20%
Severe Problems	0.00%	0.63%	0.00%	0.82%	0.00%	0.88%	4.17%
Unable to Wash	0.00%	0.00%	0.00%	1.64%	0.49%	0.00%	0.75%
**Usual Activities**							
No Problems	88.00%	76.53%	60.71%	57.38%	70.87%	71.05%	51.12%
Slight Problems	8.00%	13.95%	17.86%	21.31%	15.05%	20.18%	19.08%
Moderate Problems	3.00%	8.67%	15.18%	16.39%	10.19%	5.26%	18.03%
Severe Problems	1.00%	0.00%	6.25%	3.28%	3.40%	2.63%	8.94%
Unable to do Usual	0.00%	0.85%	0.00%	1.64%	0.49%	0.88%	2.83%
**Pain & Discomfort**							
No Pain	49.00%	58.56%	36.61%	41.80%	57.28%	42.98%	29.21%
Slight Pain	22.00%	23.89%	33.04%	27.05%	19.42%	22.81%	32.49%
Moderate Pain	23.00%	13.95%	24.11%	27.05%	14.56%	30.70%	22.21%
Severe Pain	6.00%	2.96%	5.36%	3.28%	7.28%	3.51%	12.07%
Extreme Pain	0.00%	0.00%	0.00%	0.00%	0.00%	0.00%	0.00%
**Anxiety & Depression**							
Not Anxious	71.00%	53.28%	76.79%	48.36%	59.71%	53.51%	45.31%
Slightly Anxious	18.00%	28.75%	17.86%	28.69%	20.39%	39.47%	26.23%
Moderately Anxious	7.00%	14.59%	5.36%	15.57%	12.14%	4.39%	16.99%
Severely Anxious	4.00%	2.11%	0.00%	5.74%	5.83%	1.75%	8.05%
Extremely Anxious	0.00%	1.27%	0.00%	1.64%	1.94%	0.88%	3.43%
**Mean** **EQ-5D-5L Utility Score**	0.93 (0.11)	0.88 (0.18)	0.85 (0.17)	0.82 (0.20)	0.83 (0.26)	0.92 (0.13)	0.66 (0.30)

**Fig. 1:**
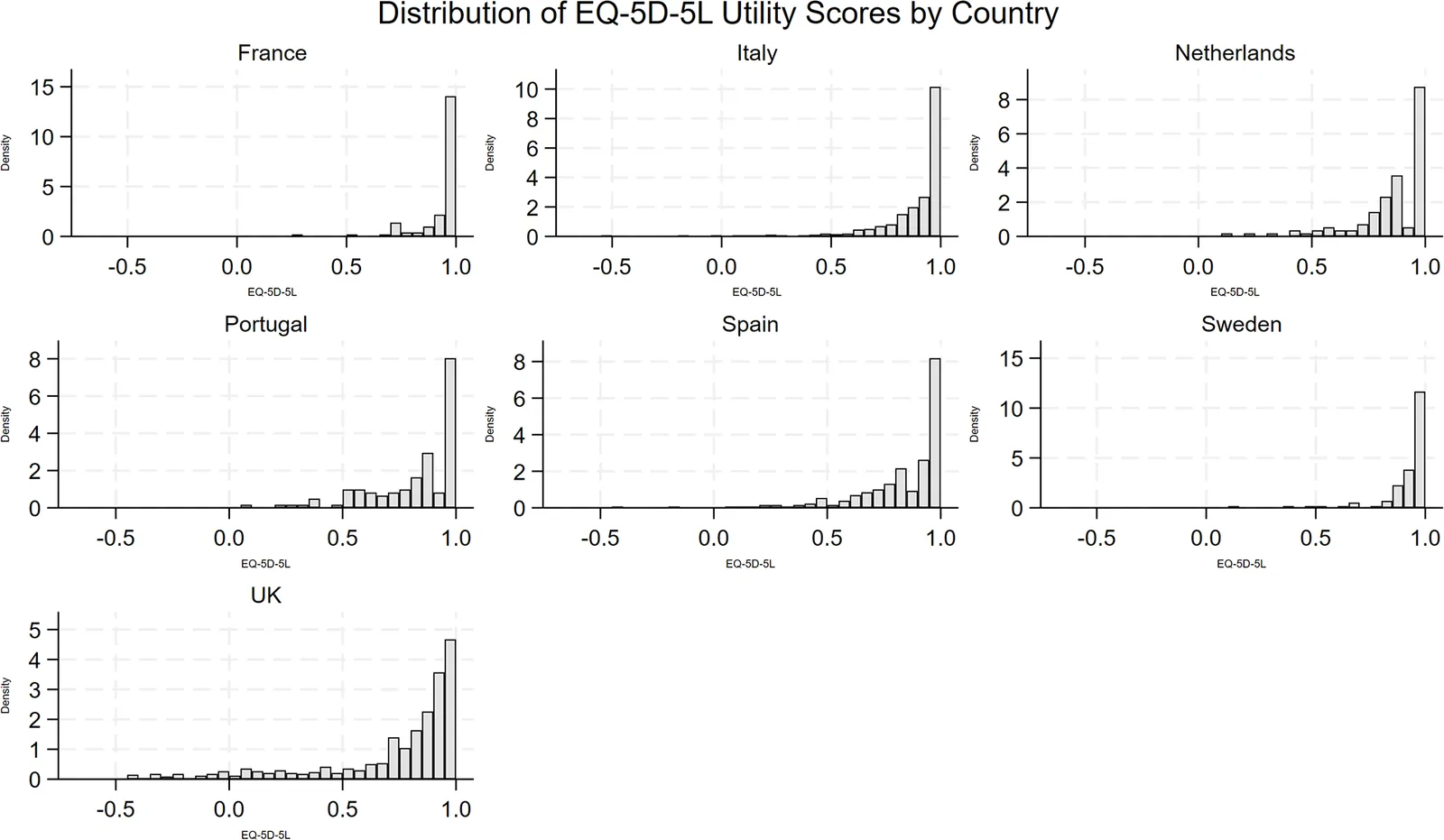
Histogram of EQ-5D-5L utility values by country

The EQ-5D-5L utility scores are not strictly comparable across countries, as each country has a separate value set. Histograms showing the distributions of the EQ-5D-5L utility values for each country are shown in Fig. 1. As shown, the French, Italian and Swedish samples had a very high proportion of respondents reporting the maximum possible utility score (1), with this proportion the lowest in the UK sample. Very few individuals reported their utility as being worse than death across the seven samples. Unlike the other samples, the UK and Portuguese samples exhibited evidence of bimodal distributions.

The responses to the NASH-CHECK for the seven samples are shown in Table 3. There are several differences between the different samples that are worth noting. The Dutch, Portuguese and UK samples reported relatively worse outcomes for almost all single (fatigue, sleep problems and itchy skin) and combined (cognitive symptoms, emotional impact and social impact) item scales. The Italian and Spanish samples were more comparable but still exhibited some small differences. The largest differences were for the sleep single item scale and the emotional and social impact scales, with the Italian respondents on average reporting worse outcomes for all these scales.

**Table 3:** NASH-CHECK responses in final estimation sample by country

	France (n = 100)	Italy (n = 473)	Netherlands (n = 112)	Portugal (n = 122)	Spain (n = 206)	weden (n = 114)	UK (n = 671)
Abdominal Pain scale	0.79 (1.73)	1.45 (2.30)	1.82 (2.61)	1.33 (2.21)	1.66 (2.39)	0.96 (1.64)	1.54 (2.50)
Abdominal Bloating scale	1.30 (2.06)	2.10 (2.77)	2.54 (3.10)	2.53 (3.16)	1.94 (2.57)	1.25 (2.16)	2.29 (2.96)
Fatigue scale	2.86 (2.59)	3.24 (2.87)	4.65 (3.08)	4.79 (3.32)	2.84 (3.25)	1.92 (2.32)	4.19 (3.15)
Sleep scale	2.07 (2.76)	2.89 (3.12)	3.38 (3.13)	3.58 (3.55)	2.30 (3.08)	1.71 (2.65)	3.86 (3.59)
Itchy Skin scale	0.86 (1.86)	1.64 (2.57)	2.40 (2.83)	2.47 (2.92)	1.58 (2.35)	1.39 (2.25)	2.54 (2.97)
Cognitive Symptoms scale	0.79 (1.13)	1.57 (2.05)	2.41 (2.21)	2.69 (2.59)	1.41 (2.13)	1.22 (1.80)	2.46 (2.60)
Activity Limitations scale	1.39 (1.82)	1.64 (1.95)	1.98 (2.26)	3.20 (2.56)	1.68 (2.02)	1.19 (1.75)	2.68 (2.78)
Emotional Impact scale	1.36 (1.87)	2.63 (2.16)	1.91 (1.92)	3.31 (2.59)	1.84 (2.10)	1.92 (2.12)	2.76 (2.63)
Social Impact scale	0.39 (1.22)	1.27 (1.86)	1.52 (2.17)	1.34 (1.84)	0.65 (1.42)	0.80 (1.34)	1.78 (2.39)

**Notes:** Mean values, standard deviations in parentheses

### Comparisons of fit statistics

The comparisons of fit statistics for the samples are shown in Tables 4-10. The main results are summarised below.

- For the French sample, the Two-part model had the lowest MAE while the Tobit model had the lowest RMSE. Of the ALDVMMs, the two-component model had the lowest AIC and BIC.
- In the Italian sample, the three-component ALDVMM model had the lowest MAE, while the Tobit model had the lowest RMSE. Of the ALDVMMs, the three-component model had the lowest AIC and BIC.
- For the Netherlands sample, the OLS model had the lowest MAE while the Two-part model had the lowest RMSE. Of the ALDVMMs, the two-component model had the lowest AIC and BIC.
- For the Portuguese sample, the Two-part model had the lowest MAE while the OLS model had the lowest RMSE. Of the ALDVMMs, the two-component model had the lowest AIC and BIC.
- In the Spanish sample, the Two-part model had the lowest MAE, and the 1-component ALDVMM had the lowest RMSE. Of the ALDVMMs, the two-component model had the lowest AIC and BIC.
- For the Swedish sample, the Two-part model had the lowest MAE while the Tobit model had the lowest RMSE.
- For the UK sample, the Tobit model had the lowest MAE and the lowest RMSE. Of the ALDVMMs, the two-component model had a lower AIC and BIC compared with the single-component model.

**Table 4:** Goodness-of-fit results in the French sample (*N* = 100)

	MAE	RMSE	AIC	BIC	Mean	Absolute Difference in Utility
**OLS**	0.0471	0.0764	N/A	N/A	**0.9322**	**0.0000**
**Tobit**	0.0470	**0.0751**	N/A	N/A	0.9272	−0.0050
**Two-Part (OLS-Logit)**	**0.0451**	0.0915	N/A	N/A	**0.9322**	**0.0000**
**ALDVMM (1 Component)**	0.0474	0.0752	−56.7845	−22.9173	0.9262	−0.0060
**ALDVMM (2 Components)**	0.0474	0.0828	**−98.7547**	**−28.4151**	0.9332	0.0010

*MAE* Mean absolute error, *RMSE* Root Mean Squared Error, *AIC* Akaike Information Criteria, *BIC* Bayesian Information Criteria. Mean observed = 0.9322

The optimal values for each column are **in bold.** The AIC and BIC cannot be compared between OLS, Tobit, Two-Part and ALDVMMs and are therefore so not included for OLS, Tobit or Two-Part.

**Table 5:** Goodness-of-fit results in the Italian sample (*N* = 473)

	MAE	RMSE	AIC	BIC	Mean	Absolute Difference in Utility
**OLS**	0.0767	0.1257	N/A	N/A	**0.8821**	**0.0000**
**Tobit**	0.0762	**0.1220**	N/A	N/A	0.8748	−0.0073
**Two-Part (OLS-Logit)**	0.0748	0.1495	N/A	N/A	**0.8821**	**0.0000**
**ALDVMM (1 Component)**	0.0769	0.1224	−87.0034	−32.9351	0.8730	−0.0091
**ALDVMM (2 Components)**	0.0740	0.1273	−270.3715	−158.0759	0.8879	0.0058
**ALDVMM (3 Components)**	**0.0746**	0.1313	**−439.1905**	**−268.6676**	0.8915	0.0094

*MAE* Mean absolute error, *RMSE* Root Mean Squared Error, *AIC* Akaike Information Criteria, *BIC* Bayesian Information Criteria. Mean observed = 0.8821

The optimal values for each column are **in bold.** The AIC and BIC cannot be compared between OLS, Tobit, Two-Part and ALDVMMs and are therefore so not included for OLS, Tobit or Two-Part.

**Table 6:** Goodness-of-fit results in the Netherlands sample (*N* = 112)

	MAE	RMSE	AIC	BIC	Mean	Absolute Difference in Utility
**OLS**	**0.0900**	0.1459	N/A	N/A	**0.8480**	**0.0000**
**Tobit**	0.0949	0.1471	N/A	N/A	0.8357	−0.0123
**Two-Part (OLS-Logit)**	0.0911	**0.1359**	N/A	N/A	**0.8480**	**0.0000**
**ALDVMM (1 Component)**	0.0932	0.1462	−47.9470	−12.6065	0.8354	−0.0126
**ALDVMM (2 Components)**	0.0926	0.1576	**−171.3920**	**−97.9925**	0.8470	−0.0010

*MAE* Mean absolute error, *RMSE* Root Mean Squared Error, *AIC* Akaike Information Criteria, *BIC* Bayesian Information Criteria. Mean observed = 0.8480

The optimal values for each column are **in bold.** The AIC and BIC cannot be compared between OLS, Tobit, Two-Part and ALDVMMs and are therefore so not included for OLS, Tobit or Two-Part.

**Table 7:** Goodness-of-fit results in the Portuguese sample (*N* = 122)

	MAE	RMSE	AIC	BIC	Mean	Absolute Difference in Utility
**OLS**	0.0905	**0.1278**	N/A	N/A	**0.8213**	**0.0000**
**Tobit**	0.0873	0.1285	N/A	N/A	0.8193	−0.0020
**Two-Part (OLS-Logit)**	**0.0830**	0.1415	N/A	N/A	**0.8213**	**0.0000**
**ALDVMM (1 Component)**	0.0878	0.1278	−35.6431	0.8092	0.8178	−0.0035
**ALDVMM (2 Components)**	0.0863	0.1334	**−86.3126**	**−10.6040**	0.8154	−0.0059

*MAE* Mean absolute error, *RMSE* Root Mean Squared Error, *AIC* Akaike Information Criteria, *BIC* Bayesian Information Criteria. Mean observed = 0.8213

The optimal values for each column are **in bold.** The AIC and BIC cannot be compared between OLS, Tobit, Two-Part and ALDVMMs and are therefore so not included for OLS, Tobit or Two-Part.

**Table 8:** Goodness-of-fit results in the Spanish sample (*N* = 206)

	MAE	RMSE	AIC	BIC	Mean	Absolute Difference in Utility
**OLS**	0.0848	0.1406	N/A	N/A	**0.8343**	**0.0000**
**Tobit**	0.0846	0.1407	N/A	N/A	0.8345	0.0002
**Two-Part (OLS-Logit)**	**0.0819**	0.1752	N/A	N/A	**0.8343**	**0.0000**
**ALDVMM (1 Component)**	0.0857	**0.1398**	23.8780	67.1404	0.8305	−0.0038
**ALDVMM (2 Components)**	0.0854	0.1450	**−28.1986**	**61.6540**	0.8321	−0.0022

*MAE* Mean absolute error, *RMSE* Root Mean Squared Error, *AIC* Akaike Information Criteria, *BIC* Bayesian Information Criteria. Mean observed = 0.8343

The optimal values for each column are **in bold.** The AIC and BIC cannot be compared between OLS, Tobit, Two-Part and ALDVMMs and are therefore so not included for OLS, Tobit or Two-Part.

**Table 9:** Goodness-of-fit results in the Swedish sample (*N* = 114)

	MAE	RMSE	AIC	BIC	Mean	Absolute Difference in Utility
**OLS**	0.0465	0.0683	N/A	N/A	**0.9213**	**0.0000**
**Tobit**	0.0430	**0.0651**	N/A	N/A	0.9149	−0.0064
**Two-Part (OLS-Logit)**	**0.0428**	0.0768	N/A	N/A	**0.9213**	**0.0000**
**ALDVMM (1 Component)**	0.0433	0.0650	−118.8542	−83.2836	0.9144	−0.0069

*MAE* Mean absolute error, *RMSE* Root Mean Squared Error, *AIC* Akaike Information Criteria, *BIC* Bayesian Information Criteria. Mean observed = 0.9213

The optimal values for each column are **in bold.** The AIC and BIC cannot be compared between OLS, Tobit, Two-Part and ALDVMMs and are therefore so not included for OLS, Tobit or Two-Part.

**Table 10:** Goodness-of-fit results in the UK sample

	MAE	RMSE	AIC	BIC	Mean	Absolute Difference in Utility
**OLS**	0.1305	0.1921	N/A	N/A	**0.7267**	**0.0001**
**Tobit**	**0.1278**	**0.1857**	N/A	N/A	0.7113	−0.0153
**Two-Part (OLS-Logit)**	0.1287	0.2098	N/A	N/A	**0.7267**	**0.0001**
**ALDVMM (1 Component)**	0.1285	0.1861	19.8164	78.4304	0.7103	−0.0163
**ALDVMM (2 Components)**	0.1327	0.2032	−223.9049	−102.1682	0.7506	0.0240

*MAE* Mean absolute error, *RMSE* Root Mean Squared Error, *AIC* Akaike Information Criteria, *BIC* Bayesian Information Criteria. Mean observed = 0.7266

The optimal values for each column are **in bold.** The AIC and BIC cannot be compared between OLS, Tobit, Two-Part and ALDVMMs and are therefore so not included for OLS, Tobit or Two-Part

### Cumulative distribution functions

**Fig. 2:**
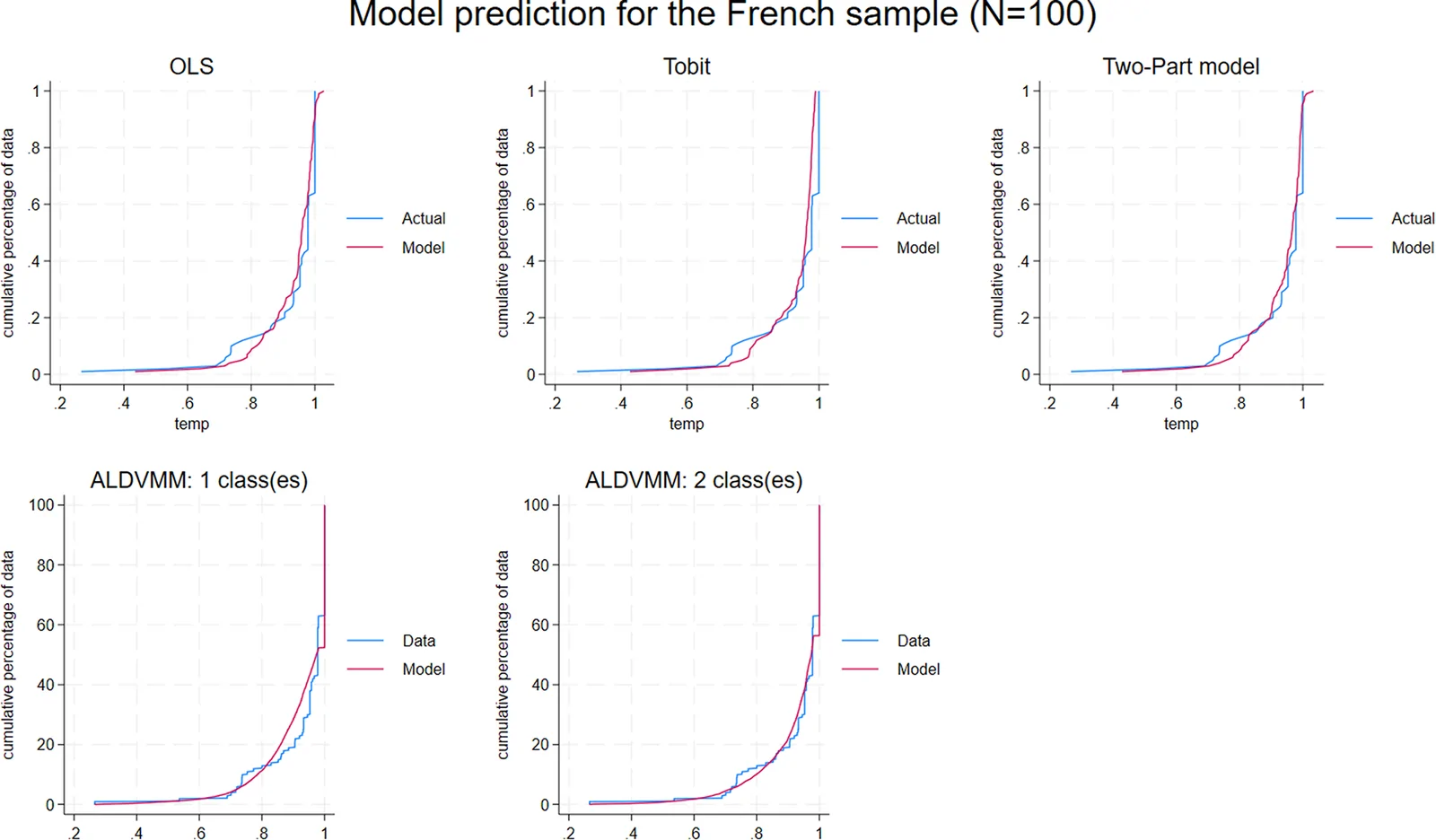
Model prediction for the French sample (*n* = 100)

**Fig. 3:**
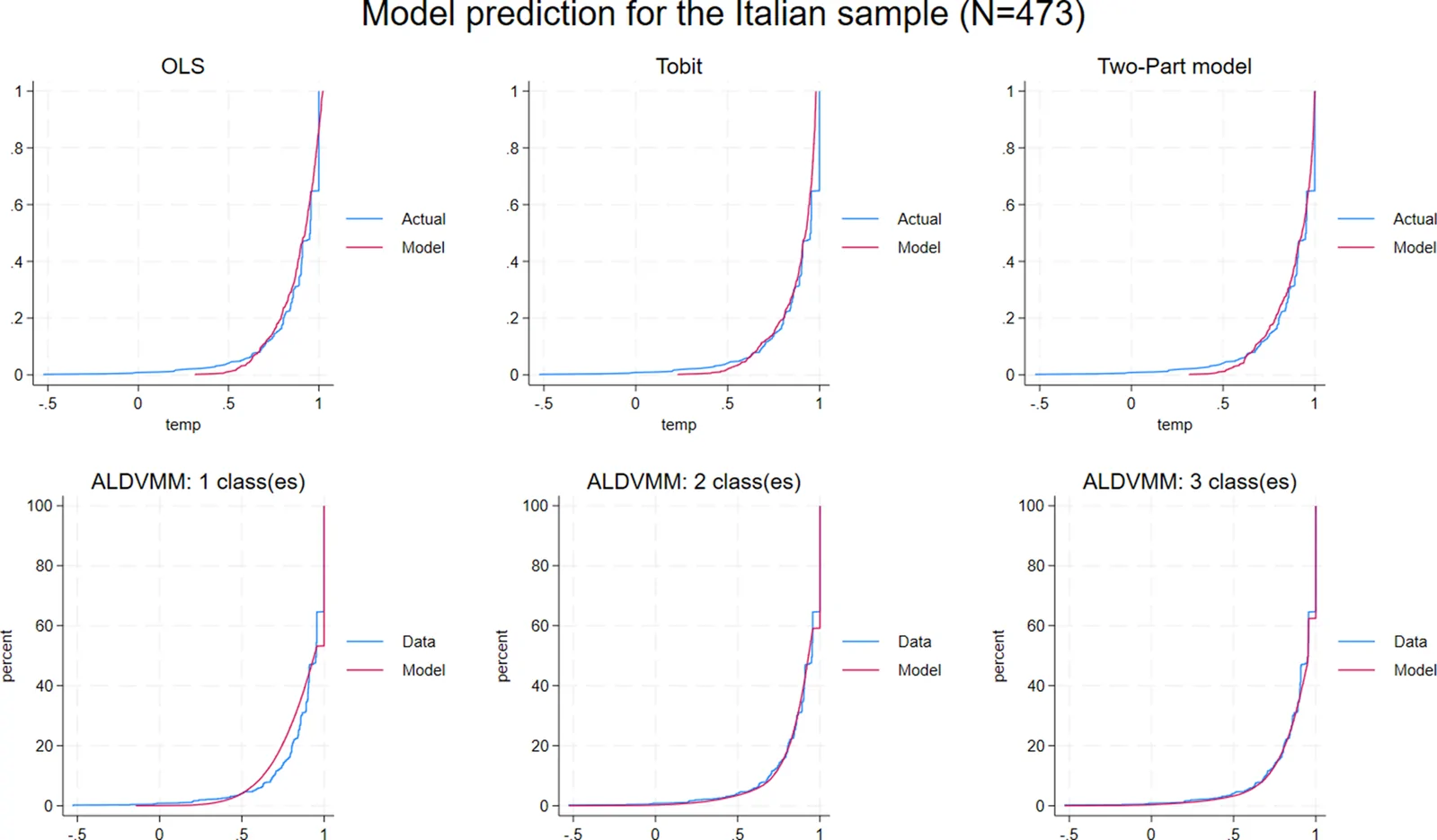
Model prediction for the Italian sample (n = 473

**Fig. 4:**
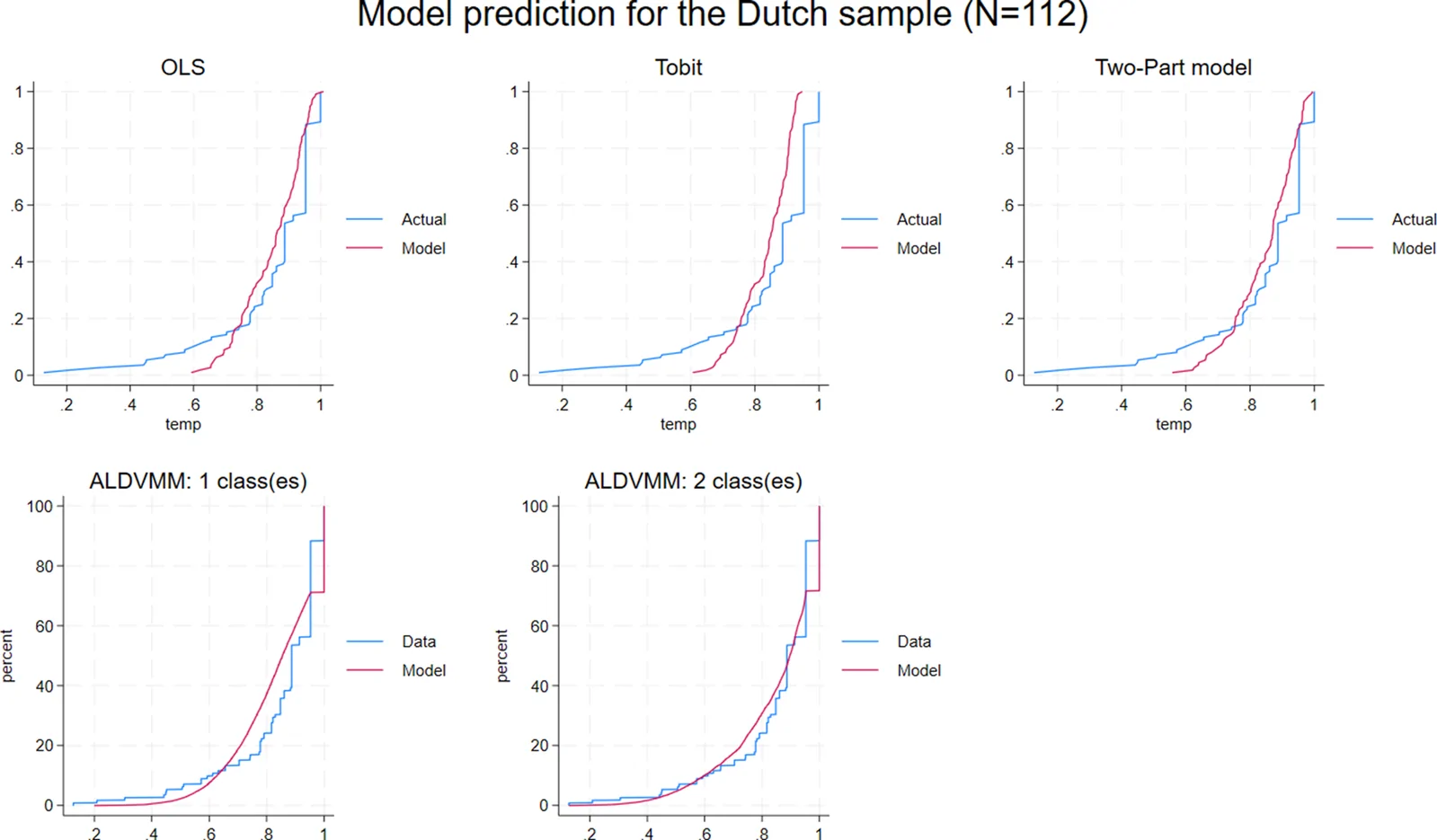
Model prediction for the Netherlands sample (*n* = 112)

**Fig. 5:**
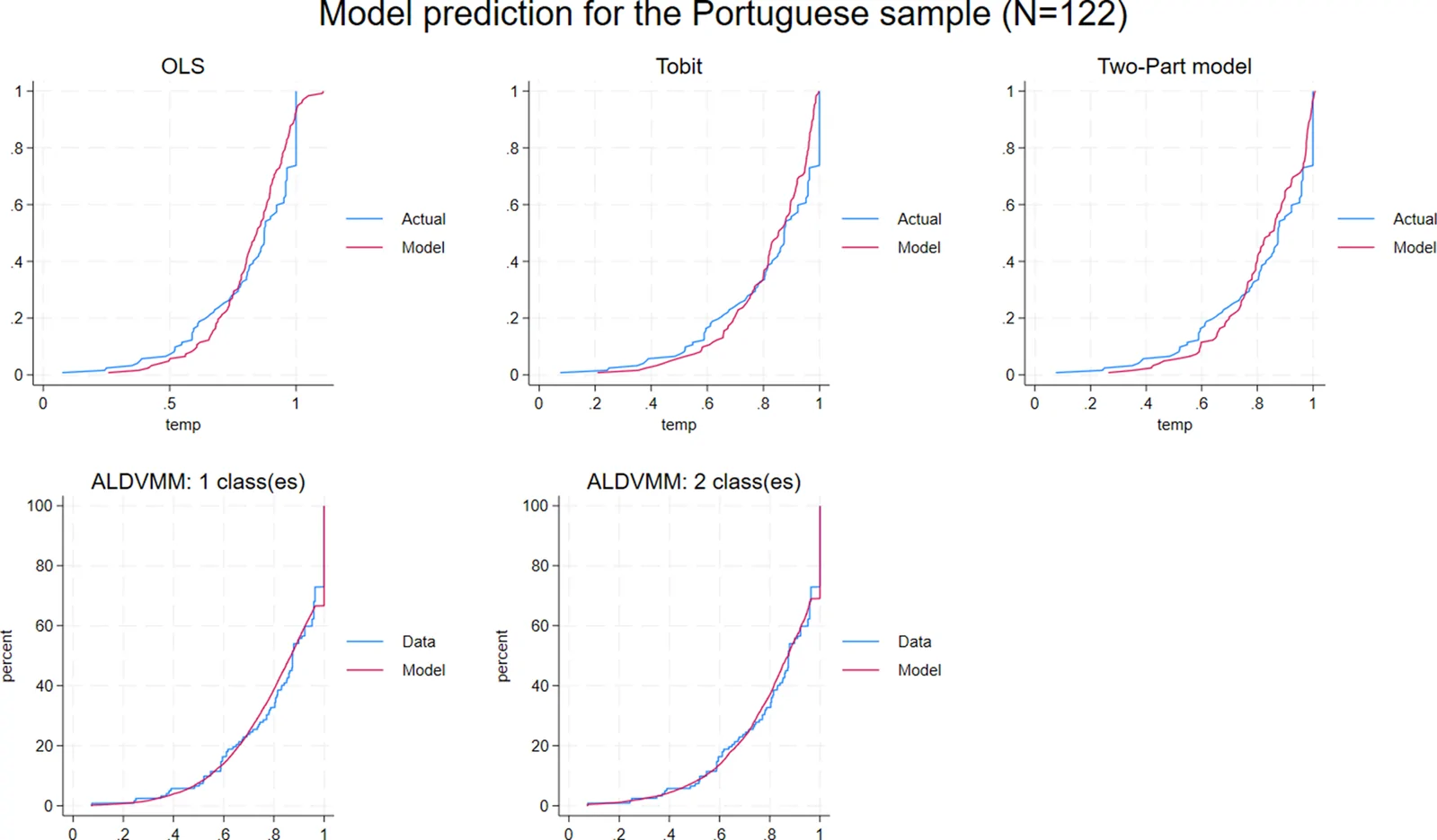
Model prediction for the Portuguese sample (*n* = 122)

**Fig. 6:**
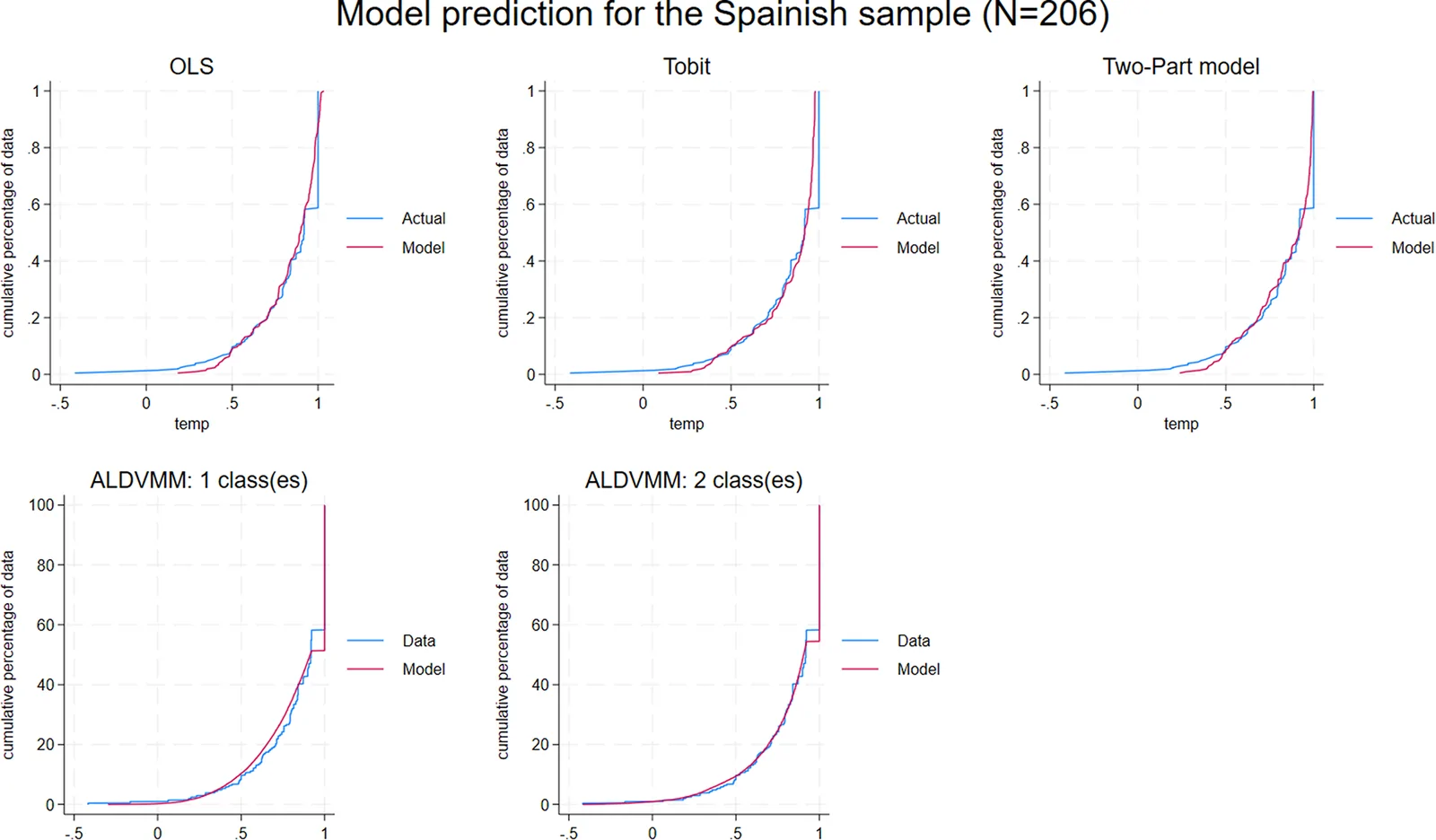
Model prediction for the Spanish sample (*n* = 206)

**Fig. 7:**
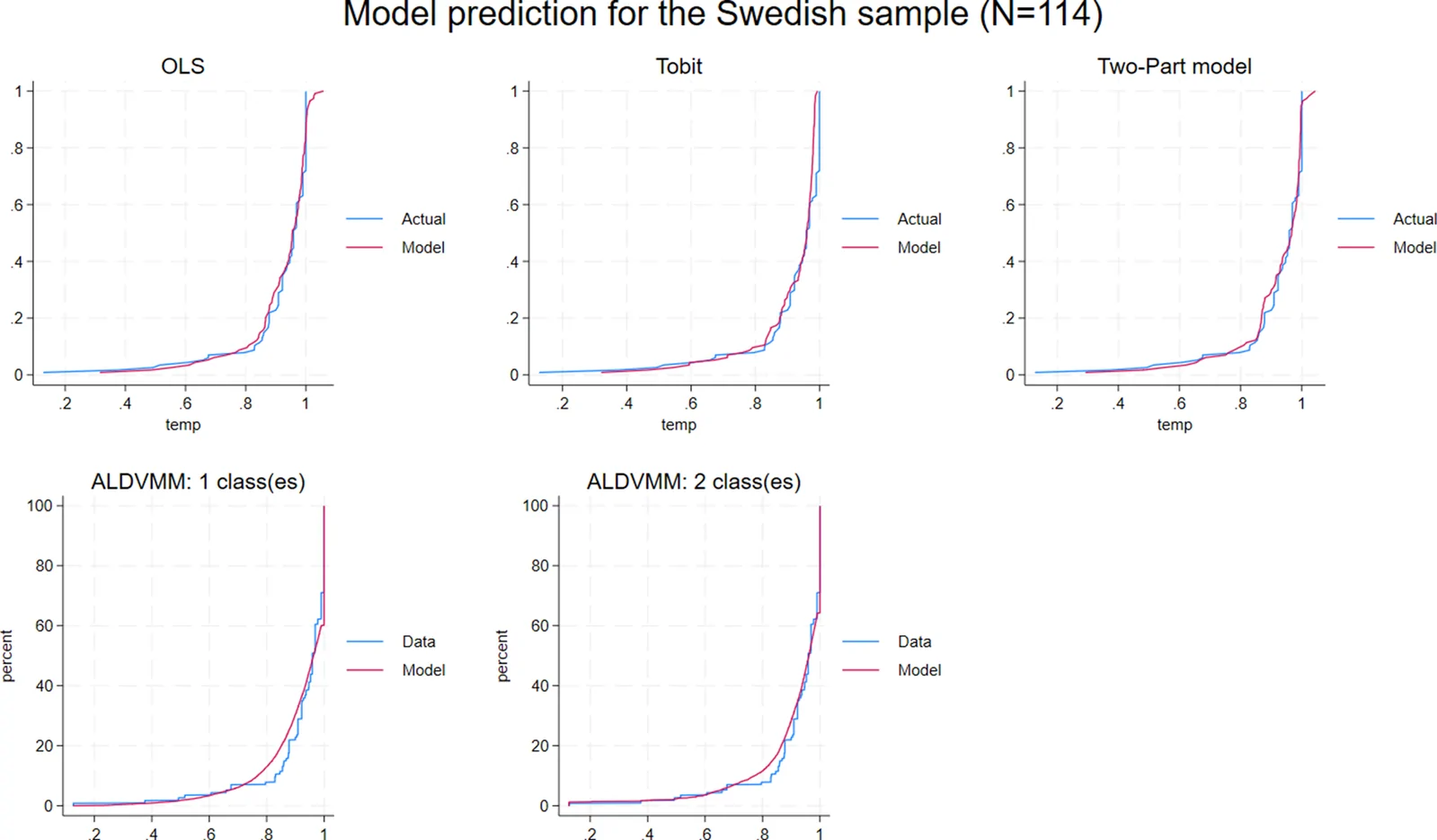
Model prediction for the Swedish sample (*n* = 114)

**Fig. 8:**
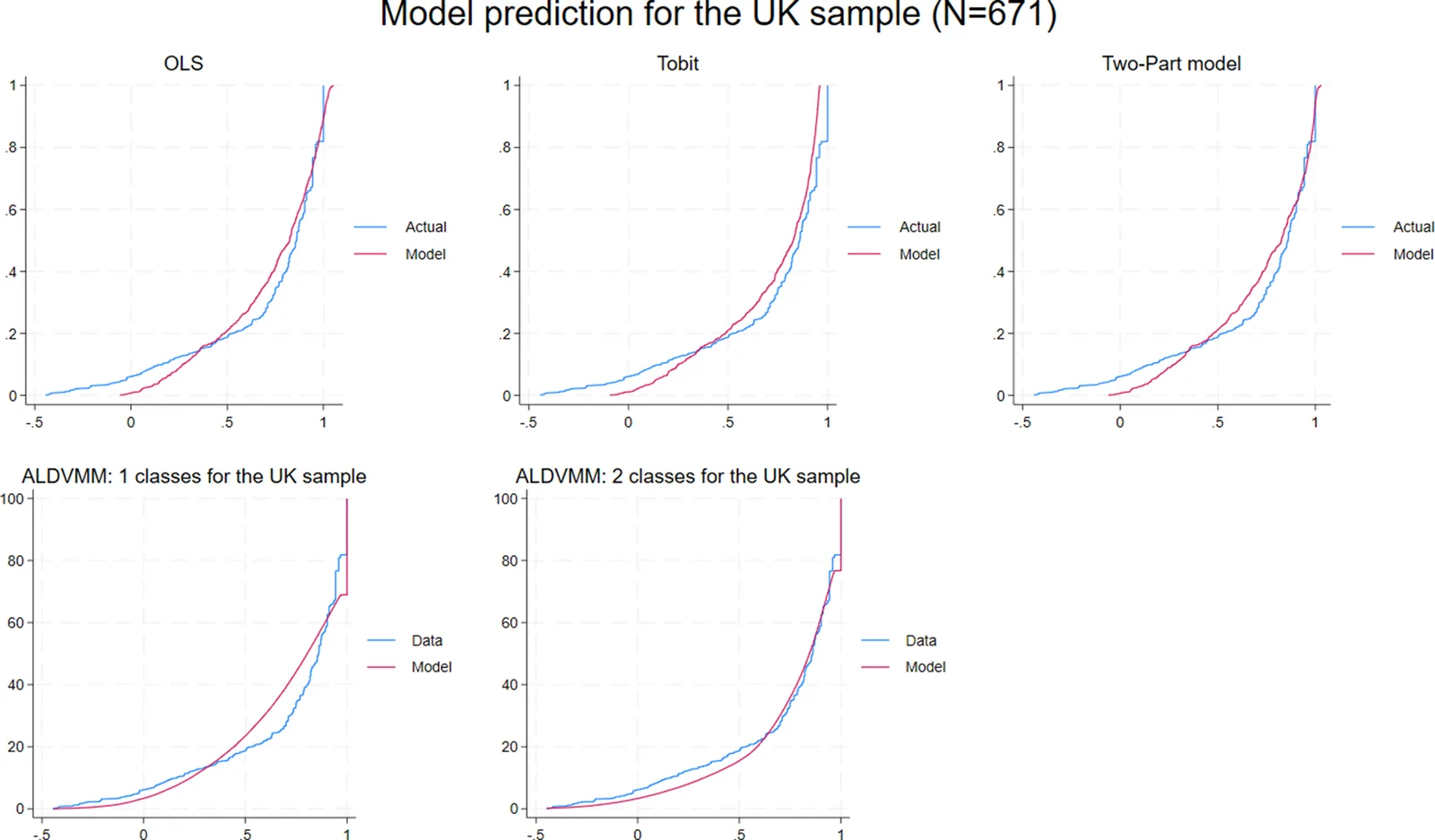
Model prediction for the Uk sample (*n* = 671)

The CDFs of the prediction models for each sample are shown in Figs. 2–8. Looking at the results for the OLS prediction models, the model underestimates and overestimates the EQ-5D-5 L utility at various points over the distribution. In particular, it underestimates the EQ-5D-5 L utility for those in fairly good health and sometimes produces unrealistic values (i.e., a utility value > 1)

Similarly to the OLS model, the Tobit and Two-Part models tended to underestimate values for fairly good health states, especially in the gap between the mass of individuals who score one and the next feasible EQ-5D-5L value. In particular, the Tobit and Two-Part models underpredict all the values above 0.8 for the Netherlands sample. As expected, the Two-Part models failed to predict health states that are “worse than dead” (i.e., negative value). Occasionally, the Two-Part models also predicted unrealistic values (i.e., a value larger than 1) at the upper end of the distribution.

The ALDVMMs generally have a better fit of observed data across the whole of the distribution. Particularly, the model preserves the attractive statistical features of other candidate models (i.e., able to cover negative values, censored at 1), whilst correctly identifying the large difference in utility between the highest utility value (i.e., 1) and the next highest utility value. As for the comparison within the ALDVMM models with different classes, it is observed that the single-component ALDVMM tend to underestimate the EQ-5D-5L utility for those in moderate health and overestimates the EQ-5D-5L utility for those in very good health. However, the models with higher number of components, fit the observed data more closely across the whole spectrum of severity. These preferred features exhibit in all country samples, even for those with relatively small sample size.

### Models selected

On the basis of the summary measures of goodness of fit and visual inspection of the CDFs, the ALDVMM models are chosen as the most appropriate model specification. Specifically, the one-component ALDVMM was chosen as the most appropraite model for the Swedish sample, the two-component ALDVMM was chosen as the most appropriate model for the French, Netherlands, Portuguese, Spanish and UK samples, and three component ALDVMM was selected for the Italian sample. A series of calculators created in Microsoft Excel are included as part of the supplementary materials. These files allow the user to calculate an EQ-5D-5L utility score from the NASH-CHECK using the algorithms generated from this study.

## Discussion

This paper provides estimates for the prediction EQ-5D-5L utility scores as a function of the NASH-CHECK for patients with MASLD in samples from seven European countries. These estimates therefore allow EQ-5D-5L utility scores to be calculated in clinical studies where the NASH-CHECK, but not the EQ-5D-5L, has been collected in these countries. In line with a number of previous studies, we find that although standard regression models used in mapping analyses (such as OLS, Tobit or Two-Part estimators) may appear good in terms of certain summary statistics (such as MAE and RMSE), these summary measures fail to consider how any one model may perform over the full distribution. For example, our analysis showed that the OLS models sometimes underestimate EQ-5D-5L utility for those in good health. This is a common feature of OLS models in mapping analyses and has been shown in a number of previous studies [31, 32]. Aided by visual plots of the cumulative distribution of predicted values and observed values, the ALDVMMs appear to fit the data better over the whole of the distribution for all the samples and therefore seem better equipped at predicting the EQ-5D-5L compared to other models. Similar results are observed in other mapping studies using the ALDVMM model [33, 34].

An alternative mixture model that has been used in recent mapping studies is the beta mixture model [35, 36]. Similar to the ALDVMM model, the beta mixture model combines finite mixture of distributions, allowing the joint distribution to exhibit nonparametric features as the number of individual components increases. The model can incorporate features that are absent in the standard beta distribution, such as multimodality. However, compared to the ALDVMM, the beta mixture model is sensitive to large number of observations at the boundary, e.g., at value 1 which can be a particular feature of the EQ-5D. This model can also not reflect the gap between a score of one (full health) and the next value commonly found in EQ-5D data. These disadvantages are overcome by the ALDVMM model.

To maintain consistency, we used the same set of variables as covariates across all models for all the samples; these were all NASH-CHECK dimensions, age and gender. Although using different explanatory variables is not uncommon in mapping studies, some studies have formalised the variable selection using machine learning techniques such as the Least Absolute Shrinkage and Selection Operator (LASSO) [37–39]. These algorithms can incorporate a large number of variables in a linear or non-linear pattern to improve prediction accuracy. However, there is currently limited guidance on best practice for using machine learning in mapping, and studies which have used these methods have yielded mixed results [37–39].

Several shortcomings with this study should be considered. The principal limitation with our analyses is that the dataset used (the SLD registry) contains patients with MASLD, rather than patients confirmed to have MASH. As noted previously, the NASH-CHECK was explicitly developed and validated for patients with cirrhotic MASH and was not validated in a wider MASLD population. It is likely that those with MASLD will, on average, be healthier than those patients confirmed to have MASH. However, whilst the NASH-CHECK is currently not validated in this population, it is anticipated that it will be acceptable for this population. For example, a number of patients with early-stage MASH (Fibrosis Level F1) were included in the qualitative content generation work which underpinned the development of the classification system of the measure [5]. Furthermore, recent qualitative work was explicitly conducted to determine whether the NASH-CHECK is applicable for use in patients with compensated cirrhotic MASH as well as non-cirrhotic MASH. This work showed that it was and that there was the potential for the NASH-CHECK to be used in a wider population [7], With respect to relationship between specific NASH-CHECK ident and the EQ-5D-5L, one recent study in a UK MASLD population has actually shown very limited differences in EQ-5D-5L utility scores between those with cirrhosis and those with either non-advanced or advanced fibrosis [40]. Given this, we consider that it is likely to be small differences in the relationship between the NASH-CHECK and EQ-5D-5L in the two populations, however identifying the specific strength and direction of these differences will require further research.

A second limitation is that the sample sizes for French, Dutch, Portuguese, Spanish and Swedish samples are below 400, which can be considered small compared to other mapping studies in the literature. For instance, in a 2013 review of studies mapping from clinical measures to the EQ-5D, the median sample size was 1,167 [41], while a more recent review found that only 4% of mapping functions used a sample size less than 100 [42]. The issue of small sample size is likely to have contributed to some of the multi-component ALDVMMs not converging. It is worth noting that another option would have been to run a pooled model with data from multiple countries and include country fixed effects to account for heterogeneity between different countries; an approach taken by Chalet et al. (2023) [43] in a similar context. However, whilst the goal of the Chalet et al. (2023) study was to develop a mapping algorithm from a UK perspective, the goal of this study was to estimate separate algorithms in seven separate countries. In Appendix 1, we have included a table showing the results of running pooled models using the UK EQ-5D-5L value set, along with corresponding CDFs. As expected, given the increased sample size, there are better fit characteristics for the combined model compared to the country specific models, with the ALDVMM also able to be run with more components (four).

In order to maximise the available sample for analysis in each country we included repeated measures from individuals. Although we clustered the standard errors at the individual level to partially account for these repeated measures, it is worth noting that this may introduce further bias into our estimates through unobserved individual level heterogeneity or within-individual correlation over time. Related to this issue, the relatively small sample size for a mapping study meant that response-mapping methods (for example through an Ordered Logit regression model) could not be used. Therefore, for countries with small samples, this study can be used as a base for future mapping studies should larger sample sizes be available in the future.

Thirdly, as this is the first mapping study in this clinical area to our knowledge, no external dataset was available for use. Future research could test the algorithms generated as part of this study on an external dataset to ensure their external validity. Finally, mapping can be seen as a ‘second-best’ method for estimating utility scores for a non-preference based HRQoL instrument. Directly assigning the utility values to NASH-CHECK health states through a valuation study (for example through a Discrete Choice Experiment or Time Trade Off task) is considered the ‘gold standard’ method [44], however such valuation studies can be resource intensive.

## Conclusion

This study has used econometric methods to estimate mapping algorithms from the newly developed NASH-CHECK PROM to the EQ-5D-5 L in seven European countries. Our results show that it is possible to predict EQ-5D-5 L utility scores in all these countries included in this analysis, with the best results obtained when using the ALDVMM developed by Hernandez-Alvara & Wailoo (2015) [29]. The algorithms generated from these models can be used to indirectly generate EQ-5D-5 L utility scores from the NASH-CHECK in these countries, and can therefore facilitate the calculation of QALYs for use in future economic evaluations in the area of MASLD when a preference-based measure such as the EQ-5D-5 L has not been collected .

## Electronic supplementary material

Below is the link to the electronic supplementary material.

Supplementary Material 1

Supplementary Material 2

## Data Availability

This study used data from the European NAFLD Registry.

## References

[1] Anstee QM, Reeves HL, Kotsiliti E, Govaere O, Heikenwalder M. From NASH to HCC: current concepts and future challenges. NatRev Gastroenterol Hepatol. 2019;16(7):411–28. 10.1038/s41575-019-0145-7 31028350

[2] Rinella ME, Lazarus JV, Ratziu V, Francque SM, Sanyal AJ, Kanwal F, et al. A multisociety Delphi consensus statement on new fatty liver disease nomenclature. Hepatology. 2023;78(6):1966–86. 10.1097/HEP.0000000000000520. PMC10653297 37363821

[3] McPherson S, Hardy T, Henderson E, Burt AD, Day CP, Anstee QM. Evidence of NAFLD progression from steatosis to fibrosing-steatohepatitis using paired biopsies: implications for prognosis and clinical management. J Hepatol. 2015;62(5):1148–55. 10.1016/j.jhep.2014.11.034. 25477264

[4] Estes C, Anstee QM, Arias-Loste MT, Bantel H, Bellentani S, Caballeria J, et al. Modeling NAFLD disease burden in China, France, Germany, Italy, Japan, Spain, United Kingdom, and United States for the period 2016–30. J Hepatol. 2018;69(4):896-904. 10.1016/j.jhep.2018.05.036 29886156

[5] Younossi ZM, Wong G, Anstee QM, Henry L. The global burden of liver disease. Clin Gastroenterol Hepatol. 2023;21(8):1978–91. 10.1016/j.cgh.2023.04.015. 37121527

[6] Doward LC, Balp MM, Twiss J, Slota C, Cryer D, Brass CA, et al. Development of a patient-reported outcome measure for non-alcoholic steatohepatitis (NASH-CHECK): results of a qualitative study. The patient-patient-centred outcomes research. 2020;1–11. 10.1007/s40271-020-00485-w PMC8357766 33336323

[7] Twiss J, Whalley D, Doward L, Balp MM, Brass CA, Cryer D, et al. Validation of NASH-CHECK: a novel patient-reported outcome measure for non-alcoholic steatohepatitis. J Patient Rep Outcomes. 2023;7(1):1-14. 10.1186/s41687-023-00589-5. PMC10349018 37450086

[8] Doward LC, Breckons M, Balp MM, Twiss J, Vale L, McSweeney L, et al. NASH-CHECK patient-reported outcome instrument: evaluation of content and face validity for patients with metabolic dysfunction–associated steatohepatitis and compensated cirrhosis. J Patient Rep Outcomes. 2025;9(1):76. 10.1186/s41687-025-00881-6. PMC12214096 40591168

[9] Neumann PJ, Goldie SJ, Weinstein MC. Preference-based measures in economic evaluation in health care. Annu Rev Public Health. 2000;21(1):587–611. 10.1146/annurev.publhealth.21.1.587. 10884966

[10] Lambe T, Frew E, Ives NJ, Woolley RL, Cummins C, Brettell EA, et al. Mapping the paediatric quality of life inventory (PedsQL™) generic core scales onto the child health utility index–9 dimension (CHU-9D) score for economic evaluation in children. Pharmacoeconomics. 2018;36(4):451–65. 10.1007/s40273-017-0600-7. 29264866

[11] Dakin H, Abel L, Burns R, Yang Y. Review and critical appraisal of studies mapping from quality of life or clinical measures to EQ-5D: an online database and application of the MAPS statement. Health Qual Life Outcomes. 2018;16:13. 10.1186/s12955-018-0857-3. PMC5810002 29433510

[12] Petrou S, Rivero-Arias O, Dakin H, Longworth L, Oppe M, Froud R, et al. Preferred reporting items for studies mapping onto preference-based outcome measures: the MAPS statement. Int J Technol Assess Health Care. 2015;31(4):230–35. 10.1017/S0266462315000379. PMC4688917 26235547

[13] Wailoo AJ, Hernandez-Alava M, Manca A, Mejia A, Ray J, Crawford B, et al. Mapping to estimate health-state utility from non–preference-Based outcome measures: an ISPOR good practices for outcomes Research task force report. Value Health. 2017;20(1):18–27. 10.1016/j.jval.2016.11.006. 28212961

[14] Hardy T, Wonders K, Younes R, Aithal GP, Aller R, Allison M, et al. The European NAFLD Registry: a real-world longitudinal cohort study of non-alcoholic fatty liver disease. Contemp Clin Trials. 2020;98:106175. 10.1016/j.cct.2020.106175. 33045403

[15] Vali Y, Lee J, Boursier J, Petta S, Wonders K, Tiniakos D, et al. Biomarkers for staging fibrosis and non-alcoholic steatohepatitis in non-alcoholic fatty liver disease (the LITMUS project): a comparative diagnostic accuracy study. Lancet Gastroenterol Hepatol. 2023;8(8):714–25. 10.1016/S2468-1253(23)00017-1. 36958367

[16] https://lifesciences.rws.com/nash-check/instrument-information/instrument-scoring.

[17] Herdman M, Gudex C, Lloyd A, Janssen MF, Kind P, Parkin D, et al. Development and preliminary testing of the new five-level version of EQ-5D (EQ-5D-5L). Qual Life Res. 2011;20(10):1727–36. 10.1007/s11136-011-9903-x. PMC3220807 21479777

[18] Andrade LF, Ludwig K, Goni JMR, Oppe M, de Pouvourville G. A French value set for the EQ-5D-5L. Pharmacoeconomics. 2020;38(4):413–25. 10.1007/s40273-019-00876-4. PMC7080328 31912325

[19] Finch AP, Meregaglia M, Ciani O, Roudijk B, Jommi C. An EQ-5D-5L value set for Italy using videoconferencing interviews and feasibility of a new mode of administration. Soc. Sci. Med. 2022;292:114519. 10.1016/j.socscimed.2021.114519. 34736804

[20] Versteegh MM, Vermeulen KM, Evers SM, De Wit GA, Prenger R, Stolk EA. Dutch tariff for the five-level version of EQ-5D. Value Health. 2016;19(4):343–52. 10.1016/j.jval.2016.01.003. 27325326

[21] Ferreira PL, Antunes P, Ferreira LN, Pereira LN, Ramos-Goñi JM. A hybrid modelling approach for eliciting health state preferences: the Portuguese EQ-5D-5L value set. Qual Life Res. 2019;28(12):3163–75. 10.1007/s11136-019-02226-5. 31201730

[22] Ramos-Goñi JM, Craig BM, Oppe M, Ramallo-Fariña Y, Pinto-Prades JL, Luo N, et al. Handling data quality issues to estimate the Spanish EQ-5D-5L value set using a hybrid interval regression approach. Value Health. 2018;21(5):596–604. 10.1016/j.jval.2017.10.023. 29753358

[23] Sun S, Chuang LH, Sahlén KG, Lindholm L, Norström F. Estimating a social value set for EQ-5D-5L in Sweden. Health Qual Life Outcomes. 2022;20(1):167. 10.1186/s12955-022-02083-w. PMC9780618 36564844

[24] Rowen D, Mukuria C, Bray N, Carlton J, Longworth L, Meads D, et al. A UK value set for the EQ-5D-5L. Value Health. 2026. 10.1016/j.jval.2026.03.008. 41887403

[25] Gray AM, Rivero-Arias O, Clarke PM. Estimating the association between SF-12 responses and EQ-5D utility values by response mapping. Med Decis Mak. 2006;26(1):18–29. 10.1177/0272989X05284108. 16495197

[26] Mortimer D, Segal L. Comparing the incomparable? A systematic review of competing techniques for converting descriptive measures of health status into QALY-weights. Med Decis Mak. 2008;28(1):66–89. 10.1177/0272989X07309642. 18263562

[27] Tobin J. Estimation of relationships for limited dependent variables. Econometrica: J Econom Soc 1958;26(1):24–36. 10.2307/1907382.

[28] Cragg JG. Some statistical models for limited dependent variables with application to the demand for durable goods. Econometrica. 1971;39(5):829–44. 10.2307/1909582.

[29] Gray LA, Hernandez Alava M, Wailoo AJ. Mapping the EORTC QLQ-C30 to EQ-5D-3L in patients with breast cancer. BMC Cancer. 2021;21(1):1–10. 10.1186/s12885-021-08964-5. PMC8600775 34794404

[30] Hernández Alava M, Wailoo A. Fitting adjusted limited dependent variable mixture models to EQ-5D. Stata J. 2015;15(199-2018-5131):737–50. 10.1177/1536867X1501500307.

[31] Fuller GW, Hernandez M, Pallot D, Lecky F, Stevenson M, Gabbe B. Health state preference weights for the Glasgow outcome scale following traumatic brain injury: a systematic review and mapping study. Value Health. 2017;20(1):141–51. 10.1016/j.jval.2016.09.2398. PMC5339086 28212955

[32] Wailoo A, Hernández M, Philips C, Brophy S, Siebert S. Modeling health state utility values in ankylosing spondylitis: comparisons of direct and indirect methods. Value Health. 2015;18(4):425–31. 10.1016/j.jval.2015.02.016. 26091596

[33] Hernández Alava M, Wailoo A, Wolfe F, Michaud K. The relationship between EQ-5D, HAQ and pain in patients with rheumatoid arthritis. Rheumatology. 2013;52(5):944–50. 10.1093/rheumatology/kes400. PMC3630395 23339232

[34] Franklin M, Hernández Alava M. Enabling QALY estimation in mental health trials and care settings: mapping from the PHQ-9 and GAD-7 to the ReQoL-UI or EQ-5D-5L using mixture models. Qual Life Res. 2023;32(10):2763–78. 10.1007/s11136-023-03443-9. PMC10474206 37314661

[35] Gray LA, Wailoo AJ, Alava MH. Mapping the FACT-B instrument to EQ-5D-3L in patients with breast cancer using adjusted limited dependent variable mixture models versus response mapping. Value Health. 2018;21(12):1399–405. 10.1016/j.jval.2018.06.006. PMC6288064 30502783

[36] Gray LA, Alava MH. A command for fitting mixture regression models for bounded dependent variables using the beta distribution. Stata J Promoting Commun Stat Stata. 2018;18(1):51–75. ISSN 1536-867X 10.1177/1536867X1801800105.

[37] Hernández Alava, Wailoo A, Pudney S, Gray L, Manca A, Hernández Alava M. Mapping clinical outcomes to generic preference-based outcome measures: development and comparison of methods. Health Technol Assess. 2020;24(34):1–68. 10.3310/hta24340. PMC7357250 32613941

[38] Aghdaee M, Parkinson B, Sinha K, Gu Y, Sharma R, Olin E, et al. An examination of machine learning to map non-preference based patient reported outcome measures to health state utility values. Health Econ. 2022, Aug;31(8):1525–57. 10.1002/hec.4503. PMC9545032 35704682

[39] Zou H. The adaptive lasso and its oracle properties. J Am Stat Assoc. 2006;101(476):1418–29. 10.1198/016214506000000735.

[40] Vasquez MM, Hu C, Roe DJ, Chen Z, Halonen M, Guerra S. Least absolute shrinkage and selection operator type methods for the identification of serum biomarkers of overweight and obesity: simulation and application. BMC Med Res Methodol. 2016;16(1):1–19. 10.1186/s12874-016-0254-8. PMC5109787 27842498

[41] Papatheodoridi M, Pallini G, Aithal G, Lim HK, Cobbold J, Torres MCP, et al. Health-related quality of life in patients with nonalcoholic fatty liver disease: a prospective multi-center Uk study. Clin Gastroenterol Hepatol. 2023;21(12):3107–14.e3. 10.1016/j.cgh.2023.04.018. 37880933

[42] Dakin H. Review of studies mapping from quality of life or clinical measures to EQ-5D: an online database. Health Qual Life Outcomes. 2013;11(1):1-6 10.1186/1477-7525-11-151. PMC3844400 24010873

[43] Mukuria C, Rowen D, Harnan S, Rawdin A, Wong R, Ara R, et al. An updated systematic review of studies mapping (or cross-walking) measures of health-related quality of life to generic preference-based measures to generate utility values. Appl Health Econ Health Policy. 2019;17(3):295–313. 10.1007/s40258-019-00467-6. 30945127

[44] Chalet FX, Bujaroska T, Germeni E, Ghandri N, Maddalena ET, Modi K, et al. Mapping the insomnia severity index instrument to EQ-5D health state utilities: a United Kingdom perspective. Pharmacoeconomics Open. 2023;7(1):149–61. 10.1007/s41669-023-00388-0. PMC9928998 36703022

[45] Brazier J, Ratcliffe J, Saloman J, A. Tsuchiya measuring and valuing health benefits for economic evaluation. 2nd. Oxford: Oxford University Press; 2017.

