# Supplementary material for: Mapping the NASH-CHECK to the EQ-5D-5L in a MASLD population in seven European countries
Source: Health Qual Life Outcomes. 2026 Aug 12;24:148. doi: 10.1186/s12955-026-02554-4 (PMC13746389; doi:10.1186/s12955-026-02554-4)
Supplement: Supplementary file 1 [file 12955_2026_2554_MOESM1_ESM.docx]

| **Appendix 1 – Goodness-of-fit results for combined model (UK Value Set)** | | | | | | |
| --- | --- | --- | --- | --- | --- | --- |
|  | **MAE** | **RMSE** | **AIC** | **BIC** | **Mean** | **Absolute Difference in Utility** |
| **OLS** | 0.1064 | 0.1678 | N/A | N/A | **0.8164** | **0.0000** |
| **Tobit** | 0.1054 | **0.1631** | N/A | N/A | 0.8036 | -0.0127 |
| **Two-Part (OLS-Logit)** | 0.1045 | 0.1937 | N/A | N/A | 0.8164 | 0.0000 |
| **ALDVMM (1 Component)** | 0.1062 | 0.1634 | 83.1660 | 154.5936 | 0.8022 | -0.0142 |
| **ALDVMM (2 Components)** | 0.1046 | 0.1721 | 83.1660 | 154.5936 | 0.8313 | 0.0149 |
| **ALDVMM (3 Components)** | 0.1047 | 0.1734 | -629.7127 | -481.3631 | 0.8325 | 0.0161 |
| **ALDVMM (4 Components)** | **0.1043** | 0.1732 | **-728.3892** | **-503.1176** | 0.8307 | 0.0143 |
| *MAE* Mean absolute error, *RMSE* Root Mean Squared Error, *AIC* Akaike Information Criteria, *BIC* Bayesian Information Criteria. Mean observed = 0.8164. The optimal values for each column are **in bold.**  The AIC and BIC cannot be compared between OLS, Tobit, Two-Part and ALDVMMs and are therefore so not included for OLS, Tobit or Two-Part. N=1,789 | | | | | | |
